# Hypolipidemic and Antithrombotic Effect of 6′-*O*-Caffeoylarbutin from *Vaccinium dunalianum* Based on Zebrafish Model, Network Pharmacology, and Molecular Docking

**DOI:** 10.3390/molecules29040780

**Published:** 2024-02-08

**Authors:** Boxiao Wu, Churan Li, Huan Kan, Yingjun Zhang, Xiaoping Rao, Yun Liu, Ping Zhao

**Affiliations:** 1 Key Laboratory of Forest Resources Conservation and Utilization in the Southwest Mountains of China Ministry of Education, Southwest Forestry University, Kunming 650224, China; wbx1437@swfu.edu.cn (B.W.); lcr1545@outlook.com (C.L.); kanhuan@swfu.edu.cn (H.K.); 2 Key Laboratory of State Forestry and Grassland Administration on Highly-Efficient Utilization of Forestry Biomass Resources in Southwest China, Southwest Forestry University, Kunming 650224, China; 3 State Key Laboratory of Phytochemistry and Plant Resources in West China, Kunming Institute of Botany, Chinese Academy of Sciences, Kunming 650224, China; zhangyj@mail.kib.ac.cn; 4 Academy of Advanced Carbon Conversion Technology, Huaqiao University, Xiamen 361021, China; raoxp@hqu.edu.cn

**Keywords:** 6′-*O*-Caffeoylarbutin, *Vaccinium dunalianum* Wight, hypolipidemic, antithrombotic, network pharmacology, molecular docking

## Abstract

*Vaccinium dunalianum* leaf buds make one of the most commonly used herbal teas of the Yi people in China, which is used to treat articular rheumatism, relax tendons, and stimulates blood circulation in the body. In addition, 6′-*O*-caffeoylarbutin (CA) is a standardized extract of *V. dunalianum*, which has been found in dried leaf buds, reaching levels of up to 31.76%. Because of the uncommon phenomenon, it is suggested that CA may have a potential therapeutic role in hyperlipidemia and thrombosis. This study was designed to study the efficacy of CA on treating hyperlipidemia and thrombosis and the possible mechanisms behind these effects. Hyperlipidemia and thrombosis zebrafish models were treated with CA to observe variations of the integrated optical density within the vessels and the intensity of erythrocyte staining within the hearts. The possible mechanisms were explored using network pharmacology and molecular docking. The results demonstrate that CA exhibits an excellent hypolipidemic effect on zebrafish at concentrations ranging from 3.0 to 30.0 μg/mL and shows thrombosis inhibitory activity in zebrafish at a concentration of 30.0 μg/mL, with an inhibition rate of 44%. Moreover, network pharmacological research shows that MMP9, RELA, MMP2, PRKCA, HSP90AA1, and APP are major targets of CA for therapy of hyperlipidemia and thrombosis, and may relate to pathways in cancer, chemical carcinogenesis-receptor activation, estrogen signaling pathway, and the AGE–RAGE signaling pathway in diabetic complications.

## 1. Introduction

Hyperlipidemia is a metabolic disorder characterized by dyslipidemia and has a rapidly increasing incidence. Long-term abnormal blood lipid levels can directly cause complications, such as thrombosis, pancreatitis, and coronary heart disease, which seriously endanger human health [1,2,3]. Among the common complications of hyperlipidemia, thrombosis is one of the leading reasons for death in the elderly across the world [4,5]. Statins are commonly used to treat hyperlipidemia and thrombosis; however, their levels need to be maintained for a long time to achieve therapeutic effects, which may cause adverse events, such as myopathy or rhabdomyolysis and polyneuropathy [6,7]. Hence, the pathogenesis of hyperlipidemia and thrombosis and the development of novel and safe natural drugs have become high-interest research topics.

*Vaccinium dunalianum* Wight is an evergreen perennial of the Ericaceae family primarily located in southwest China, Bhutan, Myanmar, and Vietnam. As a traditional Chinese medicinal plant, the dried leaf buds of *V. dunalianum* are used to treat articular rheumatism, relax tendons, and stimulates blood circulation. Previous studies have suggested that *V. dunalianum* contains caffeoyl derivatives, the main compounds being 6′-*O*-caffeoylarbutin (CA), arbutin, and chlorogenic acid [8]. In addition, CA has been found to be enriched to a high degree in different parts of *V. dunalianum* and reaches levels of up to 31.76% in dried leaf buds [9]. Studies have found that CA exhibited effects like inhibiting melanin production [10], protecting the liver [11], and promoting virus resistance [12]. The medicinal value of *V. dunalianum* and its high CA content suggest that CA might have a high exploitation prospects in pharmaceutical research.

Network pharmacology is an emerging method for drug development that provides a new strategy for investigating new drugs based on proteins, genes, and drug and disease pathways [13,14]. In network pharmacology, the core concept is network targeting, which investigates the drugs–diseases relationship from the biological network perspective and clarifies the action mechanism of drugs [15]. Zebrafish lipid metabolism and circulatory system is simple and similar to that of humans in some respects, and, therefore, zebrafish hyperlipidemia and thrombus models have been widely used to assess hypolipidemic and antithrombotic activity in vivo [16,17]. In this research, the effect of CA on hyperlipidemia and thrombosis was assessed using a zebrafish model, and its mechanism of action was investigated based on Gene Ontology (GO), Kyoto Encyclopedia of Genes and Genomes (KEGG), and molecular docking analyses. This study provides a research strategy for elucidating the action mechanism of CA in hyperlipidemia and thrombosis treatment and offers data support for CA as a novel hypolipidemic and antithrombotic therapeutic agent.

## 2. Results

### 2.1. Hypolipidemic Effect of CA

**Figure 1:**
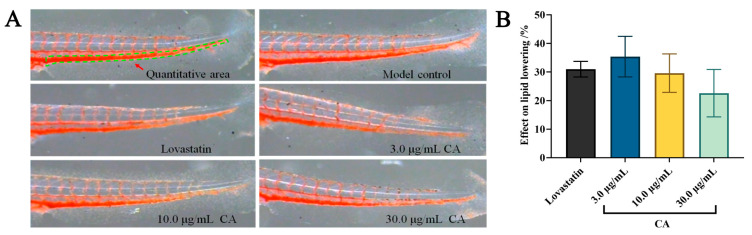
The hypolipidemic activity of CA in a zebrafish model. (**A**) Vena caudalis (green dashed line) stained with ORO in the zebrafish; (**B**) effect on lipid lowering of CA in the zebrafish.

The hypolipidemic effect of CA was detected using the Oil Red O (ORO) staining assay. The results show that both CA and lovastatin possess good inhibitory effects on egg-yolk-induced hyperlipidemia at concentrations of 3.0, 10.0, and 30.0 μg/mL (Figure 1A). The zebrafish integrated optical density (IOD) values of both the positive and CA groups were significantly less than those of model group (Table 1). In addition, the effect on lipid lowering of CA was 35 ± 7% at a concentration of 3.0 μg/mL, which was better than that of lovastatin (0.081 μg/mL) (Figure 1B and Table 1). These findings indicate that CA exerts significantly hypolipidemic effects.

### 2.2. Antithrombotic Effect of CA

**Figure 2:**
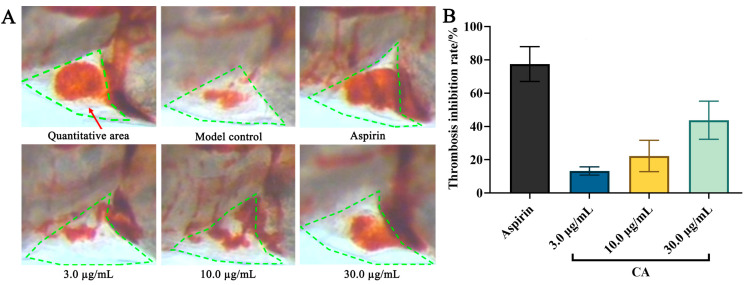
The antithrombotic activity of CA in a zebrafish model. (**A**) Cardiac red blood cells (green dashed line) stained with dianisidine in the zebrafish. (**B**) Thrombosis inhibition rate of CA in the zebrafish. All data are represented by their mean ± SE.

The antithrombotic effect of CA was investigated using the dianisidine staining assay. As shown in Figure 2 and Table 2, the intensity of erythrocyte staining (IES) of the positive control and CA groups were significantly greater than the model group. The inhibition value of CA on arachidonic acid (AA)-induced thrombosis in zebrafish was 44 ± 12% at a concentration of 30.0 μg/mL, which indicates that CA possesses a preventive effect on thrombosis.

### 2.3. Intersection Targets of CA, Hyperlipidemia, and Thrombosis

**Figure 3:**
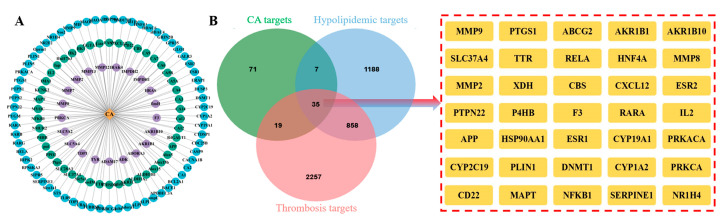
Interaction target analysis. (**A**) “CA-Target” network; (**B**) Venn diagram and intersection targets of CA, hyperlipidemia, and thrombosis.

As shown in Figure 3A, 132 bioactive targets for CA, 2088 hyperlipidemia targets, and 3169 thrombosis targets were identified based on a network computational prediction approach. The corresponding target genes were then converted to “Gene Symbol” using the UniProt database and 35 human-derived hyperlipidemia and thrombosis intersection targets were obtained after removing duplicate targets (Figure 3B).

### 2.4. Protein–Protein Interaction (PPI) Network Analysis

**Figure 4:**
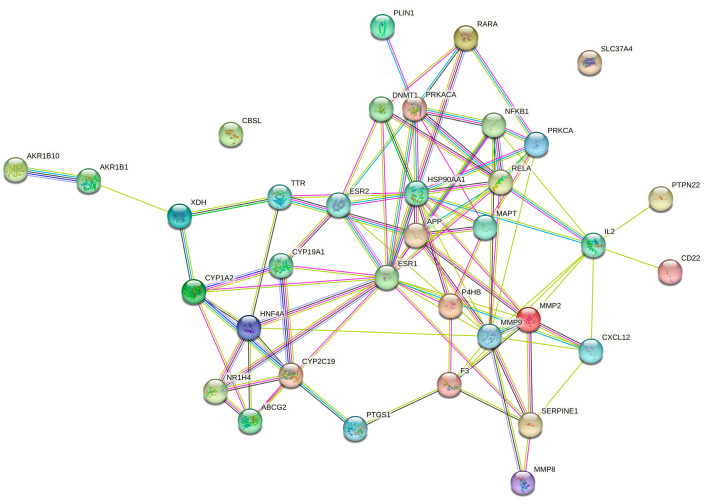
The PPI network of the 35 CA, hyperlipidemia, and thrombosis intersection targets.

A PPI network for CA, hyperlipidemia, and thrombosis with 35 nodes and 96 edges was created using the STRING online server, with a minimum required interaction score greater than 0.4. As shown in Figure 4, solid circle represents target protein, the center of the dot denotes the protein structure, and the linkage of each node denotes protein homology, gene co-expression, and gene co-evolution. With the help of the “CytoNCAA” plug-in in Cytoscape v3.7.2, the top 10 targets in the hyperlipidemia and thrombosis network (ESR1, MMP9, HSP90AA1, RELA, IL2, PRKACA, APP, MMP2, HNF4A, and PRKCA) were determined according to their degree values.

### 2.5. GO Functional and KEGG Pathway Enrichment Analysis

**Figure 5:**
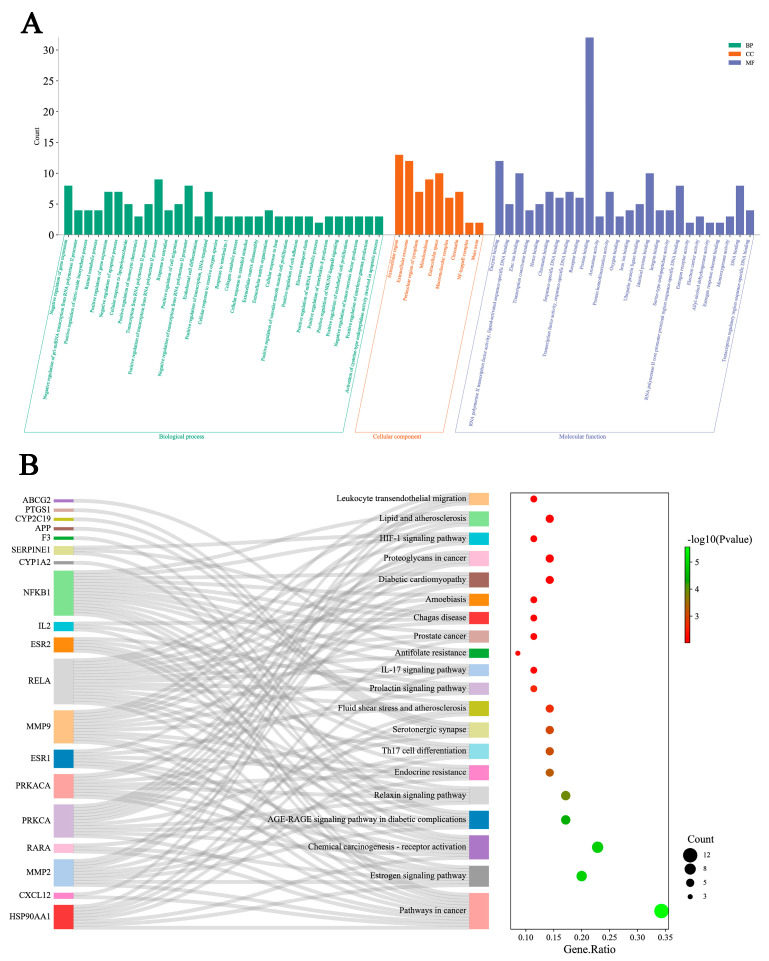
GO functional and KEGG pathway enrichment analysis. (**A**) BP, MF, and CC in the GO analysis (*p* < 0.01); (**B**) the signaling pathways identified in the KEGG pathway enrichment analysis (*p* < 0.01).

Enrichment analysis of GO functions were conducted by DAVID database with 35 intersection targets, and the results were attained after ranking according to *p* < 0.01 (Figure 5A). The GO analysis shows that the occurrence of hyperlipidemia and thrombosis involves biological processes (BP), molecular function (MF), and cellular components (CC), and CA can exert hypolipidemic and antithrombotic effects by regulating these processes.

Enrichment analysis of KEGG pathways were carried out by DAVID database with 20 pathways selected with *p* < 0.01. As shown in Figure 5B, CA is mainly used to treat hyperlipidemia and thrombosis by regulating cancer, estrogen signaling, chemical carcinogenesis-receptor activation, and AGE–RAGE signaling pathways in diabetic complications, etc. Notably, major targets, such as NFKB1, RELA, MMP9, and PRKACA, were enriched in the above pathways, which suggests possible important roles in therapy.

### 2.6. Molecular Docking and Analysis

**Figure 6:**
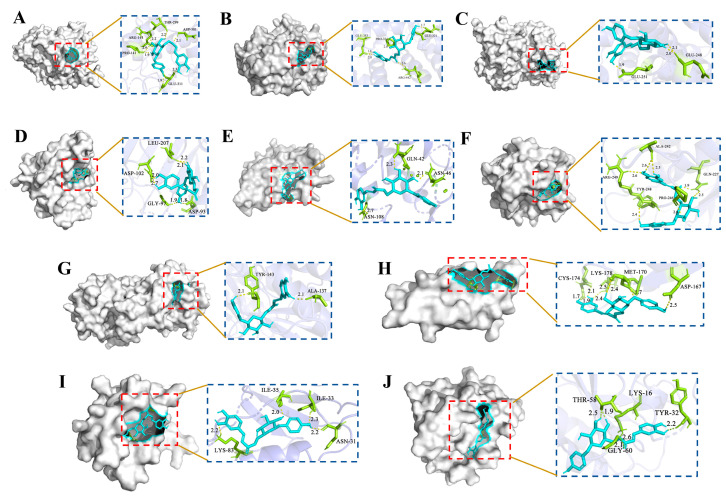
The molecular docking results of CA with core hyperlipidemia and thrombosis targets. (**A**) CA with PRKACA (PDB ID: 5M6Y); (**B**) CA with ESR1 (PDB ID: 7NFB); (**C**) CA with HNF4A (PDB ID: 8C1L); (**D**) CA with HSP90AA1 (PDB ID: 6TN5); (**E**) CA with IL2 (PDB ID: 7M2G); (**F**) CA with MMP9 (PDB ID: 6ESM); (**G**) CA with MMP2 (PDB ID: 7XJO); (**H**) CA with APP (PDB ID: 2FMA); (**I**) CA with PRKCA (PDB ID: 2GZV); (**J**) CA with RELA (PDB ID: 8ONV).

The molecular docking are commonly used to determine the most energetically favorable conformation of a small molecule ligand bound to a target protein. Generally, binding energy < −5.0 kcal/mol indicates that the target protein binds strongly to the small molecule ligand, and the lower the binding energy suggests stronger binding [18]. The re-docking analysis of the co-crystallized inhibitors to the protein targets verifies that the RMSD values for each co-crystallized inhibitors pose are below 2.00 Å, which indicates that the docking scheme is in an acceptable range of precision. The top ten targets screened based on the PPI network and CA were molecularly docked. As shown in Table 3, CA has a strong binding affinity for MMP9, RELA, MMP2, PRKCA, HSP90AA1, and APP. Additionally, the docking mode, functional groups, and protein residues of CA with hypolipidemic and antithrombotic core proteins were used for visualization by PyMol software 2.6.0 (Figure 6).

### 2.7. Calculation of ADMET-Related Properties

With the web server ADMETlab, physicochemical properties, medicinal chemistry, and ADMET properties of CA and lovastatin were calculated (Table 4). Based on the predicted results of ADMET properties, comparison to lovastatin, CA showed better performance in ADMET toxicological properties, such as hERG (hERG blockers), H-HT (human hepatotoxicity), SkinSen (skin sensitization), FDAMDD (FDA maximum daily dose), and respiratory toxicity. The predicted results demonstrate the safety of CA.

## 3. Discussion

Hyperlipidemia and thrombosis are serious chronic diseases with sudden risks that can lead to a serious decline in quality of life [19]. A long-term high-fat diet can change blood fat levels, trigger the deposition of vascular lipids on vessel walls, thicken channels, and narrow the lumen, thereby greatly increasing the risk of thrombosis [20]. Current medications for the treatment of hyperlipidemia and thrombosis are relatively homogeneous in efficacy and can cause side effects. Statin-related drugs are the leading medications for hyperlipidemia treatment, such as lovastatin, simvastatin, and resuvastatin, however, they may have a possible side effect of mild myalgia [21,22]. Compared with chemically synthesized drugs, natural products exhibit lower toxicity, higher safety levels, and wider sources. Moreover, natural products exert therapeutic effects through multi-targets and multiple pathways, providing unique advantages in the treatment of metabolic diseases [23]. Therefore, the development of new and safe hypolipidemic and antithrombotic drugs from natural products has attracted widespread attention.

*V. dunalianum* is a common herbal tea of the Yi people in China with hypolipidemic properties. The extracts of *V. dunalianum* improved high-density lipoprotein (HDL) levels and reduced triglyceride (TG), total cholesterol (TC), and low-density lipoprotein (LDL) levels in individuals with a high-fat diet [24]. In addition, an aqueous extract of *V. dunalianum* was found to improve steatosis and lower TG, TC, LDL, alanine transaminase, and aspartate transaminase levels in model rats [25]. These studies indicate that CA, the main compound in the buds of *V. dunalianum*, may have high inhibitory activity against hyperlipidemia. Although natural products such as curcumin, resveratrol, and dihydrotanshinone also exhibit hypolipidemic or antithrombotic effects [26,27,28], compared with them, CA shows a high degree of safety and is a macroscopic active ingredient in *V. dunalianum*, making it feasible to prepare in large quantities.

CA has received increasing attention as the most abundant natural product found in the buds of *V. dunalianum*. Studies have found that both the safety and melanogenesis-inhibiting activity of CA were twice as high as those of arbutin [10]. In addition, CA could exert protective effects against APAP-induced liver injury via regulating the PI3K/Akt and Nrf2 signaling pathway [11]. In this study, a zebrafish model was used to evaluate the hypolipidemic and antithrombotic activities of CA, and the potential mechanism of action was explored using network pharmacology. These results suggest that CA show a good inhibitory activity against hyperlipidemia and thrombosis, which mainly works through pathways in cancer, estrogen signaling pathway, chemical carcinogenesis-receptor activation, and the AGE–RAGE signaling pathway in diabetic complications, etc. Evidently, hyperlipidemia and cancer share some of the same hormonal metabolic pathways. Studies have found that hyperlipidemia is closely associated with cancer, and cholesterol plays an important role in the proliferation of cancer cells [29,30].

The estrogen signaling pathway is not only commonly associated with female reproduction but also exerts functions in a variety of other physiological and pathological processes, including glucose metabolism, vascular tissue repair, and lipid homeostasis [31,32]. It has been indicated that the estrogen signaling pathway is linked to hypolipidemic and antithrombotic effects [33,34]. Estrogens improve tissue lesions mainly via conjugation and activation of estrogen receptors (ERs) including the unique subtypes of ER-α and ER-β, with ER-α being the main mediator of hypolipidemic and antithrombotic effects [35,36]. Conformational shifts occur when estrogen binds to ER-α, which homodimerizes and associates with specific genetic estrogen response elements, thereby modifying related target gene expression. Studies have shown that pathway complexes can phosphorylate and activate nitric oxide synthase or other protein/enzyme systems associated with cardiovascular function despite the lack of ligands [37,38]. The estrogen signaling pathway was regulated by CA binding to HSP90AA1, MMP2, RARA, PRKACA, ESR1, MMP9, and ESR2 in this present research, which underlies the improvements observed in hyperlipidemia and thrombosis.

The AGE–RAGE signaling pathway in diabetic complications has been proven to play a key role for the pathogenesis of hyperlipidemia and thrombosis [39,40]. Prior research has shown that a high-fat diet increased the production and build-up of advanced glycosylation end products (AGE), which resulted in oxidative stress and the activation of fat storage [41,42]. NADPH oxidases can be activated by the AGE–RAGE pathway to generate active oxygen through the AGE receptor (RAGE), which resulted in the expression of redox-sensitive molecules [43,44]. The evidence demonstrates that MMP2, MMP9, and NF-κB play critical roles in the antithrombotic process [45]; moreover, MMP2 and MMP were the core targets of CA in our study.

The findings of this study reveal the potential hypolipidemic and antithrombotic mechanisms of CA, which can possibly provide a natural and cost-effective treatment for hyperlipidemia and thrombosis. However, due to the limitations of the target database, not all CA active targets have been collected. Thus, comprehensive in vitro and in vivo experiments are necessary to further investigate the pharmacological mechanism of CA in hypolipidemia and anti-thrombosis.

## 4. Materials and Methods

### 4.1. Preparation of CA and Medicine

**Figure 7:**
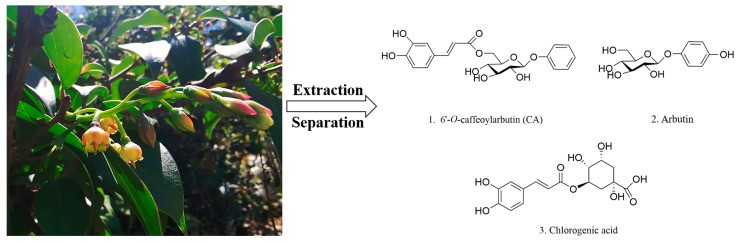
The structure formulas of main compounds in *V. dunalianum*.

*V. dunalianum* was collected from Wuding County, Yunnan Province, China. The structure formulas of main compounds, namely, CA, arbutin, and chlorogenic acid in *V. dunalianum* are shown in Figure 7. According to previously reported study, the purity of CA isolated from *V. dunalianum* was 95% [8]. AA and aspirin were purchased from Shanghai Aladdin Reagent Co., Ltd. (Shanghai, China). Lovastatin was obtained from Dalian Meilun Biological Technology Co., Ltd. (Dalian, China). CA, AA, aspirin, and lovastatin were dissolved in DMSO (Sigma, Tokyo, Japan, BCBN0845V) and subsequently diluted to their respective working concentrations.

### 4.2. Zebrafish

All experiments were performed using albino zebrafish, which were naturally pair-bred with mutant melanin alleles. All zebrafish were kept in 28 °C water with soluble salts; 200 mg/L of reverse osmosis water, pH 6.9–7.2, conductivity 480–510 μS/cm, and hardness 53.7–71.6 mg/L CaCO_3_. Feeding management complied with the international Assessment and Accreditation of Laboratory Animal Care (AAALAC) accreditation standards.

### 4.3. The Effect of CA on Hyperlipidemia and Thrombosis

After adding 0.1% egg yolk to the fish water for 48 h, the fat content in the blood of zebrafish increases rapidly and obvious lipid accumulation is visible through the naked eyes, indicating that the zebrafish hyperlipidemia model is successfully constructed [46]. In addition, after adding AA to fish water for 48 h, thrombus is generated in the trunk of zebrafish, leading to the slowing of blood flow and the reduction in blood reflux volume. When the number of cardiac erythrocytes in zebrafish heart is observed to be significantly lower than that in normal zebrafish via erythrocyte specific staining, this suggests that the zebrafish thrombus model is successfully constructed [47]. In recent years, the above methods for constructing zebrafish hyperlipidemia and thrombosis models have been widely used in the research of hypolipidemic and antithrombotic functions for natural products [48,49,50].

Specifically, 30 zebrafishes were chosen randomly and placed in 6-well culture plates for all experiments as the model group, positive group, and CA experimental group. The zebrafish hyperlipidemia model was established by adding 0.1% egg yolk to the fish water for 48 h. Subsequently, 3 mL of CA (3.0, 10.0, and 30.0 μg/mL) and lovastatin (0.081 μg/mL) was added to each experimental group. After dosing for 48 h, 10 ORO-stained zebrafishes were randomly chosen from groups, and image analysis was performed using a dissecting microscope (SZX7, Olympus, Tokyo, Japan). Image-Pro Plus 6.0 was utilized for calculating IOD within the tail vessels of the zebrafish. The effect on lipid lowing rate of CA was calculated using the following formula:

Effect on lipid lowing=[IOD (model group) −IOD (test group)]/IOD (model group)× 100%

Specifically, 30 zebrafishes were chosen randomly and placed in 6-well culture plates for all experiments as the model group, positive group, and CA experimental group. Then, 3 mL of CA (3.0, 10.0, and 30.0 μg/mL) of aspirin (22.5 μg/mL) was added to each experimental group. After administration for 3 h, AA was additionally added to induce and establish a zebrafish thrombus model. Then, 10 zebrafish were randomly picked from groups for dianisidine staining, and IES in zebrafish hearts was measured using a dissecting microscope and Image-Pro Plus 6.0 image processing software. The thrombosis inhibition rate of CA was calculated using the following equation:

Thrombosis inhibition rate=[IES (test group) − IES (model group)]/IES (model group)×100%

### 4.4. CA Target Prediction and Disease Target Identification

The SMILES string of CA was acquired from the PubChem database (https://www.ncbi.nlm.nih.gov/, accessed on 1 April 2023) and then uploaded to the SwissTargetPrediction database (http://www.swisstargetprediction.ch/, accessed on 1 April 2023), SEA database (https://sea.bkslab.org/, accessed on 1 April 2023), and TargetNet database (http://targetnet.scbdd.com/home/index/, accessed on 1 April 2023), together with the potential targets of CA-related bioactive components [51,52,53,54]. Standard gene names of potential targets were acquired from UniProt (https://beta.uniprot.org/, accessed on 1 April 2023) [55].

The keywords of “hyperlipemia”, “hypolipidemic”, “hyperlipidemial”, “thrombi”, “thrombosis”, and “thrombus” were used to search for hypolipidemic and antithrombotic targets in the GeneCards database (https://www.genecards.org/, accessed on 1 April 2023) [56], DisGeNET database (https://www.disgenet.org/, accessed on 1 April 2023) [57], and OMIM database (https://omim.org/, accessed on 1 April 2023) [58], and then a Venn diagram of CA with the above related disease targets was established.

### 4.5. PPI Network Construction and Functional Enrichment Analysis

Protein–protein interaction (PPI) data were generated from the STRING database (https://cn.string-db.org/, accessed on 2 April 2023), and PPI networks were constructed after filtering out datasets with minimum required interaction scores less than 0.4 [59,60].

GO biological processes and KEGG signaling pathways for CA targets were annotated and visualized using DAVID (http://david.abcc.ncifcrf.gov/, accessed on 2 April 2023) [61]. With the background being set to Homo sapiens, data enrichment was performed using hypergeometric test, with *p* < 0.01.

### 4.6. Molecular Docking

The key component structure files of IL2 (PDB ID: 7M2G, 1.790 A), PRKCA (PDB ID: 2GZV, 1.120 A), HSP90AA1 (PDB ID: 6TN5, 1.170 A), RELA (PDB ID: 8ONV, 1.010 A), APP (PDB ID: 2FMA, 0.850 A), PRKACA (PDB ID: 5M6Y, 1.367 A), MMP2 (PDB ID: 7XJO, 2.000 A), MMP9 (PDB ID: 6ESM, 1.104 A), ESR1 (PDB ID: 7NFB, 1.330 A), and HNF4A (PDB ID: 8C1L, 2.000 A) in SDF format were retrieved from the PubChem database (https://pubchem.ncbi.nlm.nih.gov/, accessed on 25 January 2024) [51] and converted to protein data bank (PDB) format using the OpenBabel program. The core target protein’s 3D structure was retrieved in PDB format from the PDB database (https://www.rcsb.org/, accessed on 25 January 2024) [62,63]. The co-crystal inhibitors of each protein target were re-docked to validate the reliability of the docking scheme before performing molecular docking on the new compounds. The core target protein was then dehydrated, hydrogenated, and semi-flexibly docked to the hydrogenated CA to calculate the binding energy using AutoDockTools-1.5.7 tool [64]. Visualization of the docking results were performed by PyMol software 2.6.0 to show the functional groups and protein residues at the CA-binding site of the protein [65].

### 4.7. Prediction of ADMET in Silico

The SMILES string of CA and lovastatin were acquired from the PubChem database (https://www.ncbi.nlm.nih.gov/, accessed on 4 February 2024) [51] and then uploaded to ADMETlab 2.0 predictor platform (https://admetmesh.scbdd.com/, accessed on 4 February 2024) [66] for calculation of physicochemical descriptors and prediction of the ADMET properties and drug-likeness of CA and lovastatin (absorption, distribution, excretion, and toxicity).

## 5. Conclusions

The present research results indicate that CA has dual therapeutic effects on hyperlipidemia and thrombosis. The hypolipidemic activity was better than that of lovastatin (0.081 μg/mL) at CA concentration of 3.0 μg/mL. Simultaneously, CA reduced zebrafish thrombosis at a dose of 30.0 g/mL, with an inhibition rate of 44%. Network pharmacology research showed that MMP9, RELA, MMP2, PRKCA, HSP90AA1, and APP were major targets of CA for therapy of hyperlipidemia and thrombosis and may relate to pathways in cancer, estrogen signaling pathway, chemical carcinogenesis-receptor activation, and the AGE–RAGE signaling pathway in diabetic complications, etc. This investigation demonstrates the potential for CA as a hypolipidemic and antithrombotic drug candidate, offering a basis for exploring the action mechanism. Moreover, the study provides a reference for further drug development of CA in combination with pharmacological and molecular biology assays.

## Figures and Tables

**Table 1:** Quantitative hypolipidemic activity results of CA in a zebrafish model.

Groups	Concentration (µg/mL)	IOD (Mean ± SE)	Effect on Lipid Lowering (%)
Model	-	3276 ± 51	-
Lovastatin	22.5	2259 ± 83 ***	31 ± 3
CA	3.0	2129 ± 233 ***	35 ± 7
	10.0	2310 ± 214 ***	29 ± 7
	30.0	2535 ± 273 ***	23 ± 8

*** *p* < 0.001 vs. model group.

**Table 2:** Quantitative antithrombotic activity results of CA in a zebrafish model.

Groups	Concentration (µg/mL)	IES (Mean ± SE)	Thrombosis Inhibition Rate (%)
Model	-	987 ± 94	-
Aspirin	22.5	1729 ± 99 ***	78 ± 10
CA	3.0	1102 ± 25	13 ± 3
	10.0	1191 ± 94 *	22 ± 10
	30.0	1398 ± 113 ***	44 ± 12

* *p* < 0.05 vs. model group, *** *p* < 0.001 vs. model group.

**Table 3:** The LiDockScore between CA and target proteins.

Ligand	Core Proteins Target	PDB ID	LiDockScore (kcal/mol)
CA	IL2	7M2G	−4.61
	PRKCA	2GZV	−6.11
	HSP90AA1	6TN5	−5.98
	RELA	8ONV	−6.53
	APP	2FMA	−5.23
	PRKACA	5M6Y	−4.29
	MMP2	7XJO	−6.25
	MMP9	6ESM	−7.32
	ESR1	7NFB	−4.53
	HNF4A	8O1L	−3.12

**Table 4:** Predicted values of physicochemical, medicinal chemical, and ADMET properties of CA and lovastatin.

Property	Predicted Values	
Physicochemical property	CA	lovastatin
TPSA	166.140	72.830
LogS (solubility)	−2.421	−4.665
LogD (distribution coefficient D)	1.581	4.067
LogP (distribution coefficient P)	1.013	3.414
Medicinal chemistry		
QED	0.211	0.672
SA score	3.710	4.690
Absorption		
Papp (Caco-2 permeability)	−6.257	−4.824
Pgp-inhibitor	0.001	0.998
Pgp-substrate	0.227	0.005
HIA (human intestinal absorption)	0.765	0.161
Distribution		
Plasma protein binding (PPB)	97.47%	94.28%
Volume distribution (VD)	0.420 L/kg	1.005 L/kg
Blood–brain barrier (BBB)	0.293	0.746
Elimination		
T 1/2 (half life time)	0.839	0.232
CL (clearance rate)	7.014	18.012
Toxicity		
hERG (hERG blockers)	0.020	0.388
H-HT (human hepatotoxicity)	0.042	0.966
DILI (drug-induced liver injury)	0.037	0.033
SkinSen (skin sensitization)	0.932	0.957
FDAMDD (FDA maximum daily dose)	0.021	0.970
Respiratory toxicity	0.034	0.678

## Data Availability

The data presented in this study are available on request from the corresponding author. The data are not publicly available due to privacy.

## References

[1] Bu J.H., Wu Y., Cai X.X., Jiang N., Jeyalatha M.V., Yu J.W., He X., He H., Guo Y.L., Zhang M.J. (2019). Hyperlipidemia induces meibomian gland dysfunction. Ocul. Surf..

[2] Gong X., Li X., Xia Y., Xu J.F., Li Q.Y., Zhang G.H., Li M.H. (2020). Effects of phytochemicals from plant-based functional foods on hyperlipidemia and their underpinning mechanisms. Trends Food Sci. Technol..

[3] Cho S.M., Lee H., Lee H.H., Baek J., Heo J.E., Joo H.J., Hong S.J., Kim H.C. (2021). Dyslipidemia fact sheets in Korea 2020: An analysis of nationwide population-based data. J. Lipid Atheroscler..

[4] Eitzman D.T., Westrick R.J., Xu Z., Tyson J., Ginsburg D. (2000). Hyperlipidemia promotes thrombosis after injury to atherosclerotic vessels in apolipoprotein E-deficient mice. Arterioscler. Thromb. Vasc. Biol..

[5] van Geffen J.P., Swieringa F., van Kuijk K., Tullemans B.M.E., Solari F.A., Peng B., Clemetson K.J., Farndale R.W., Dubois L.J., Sickmann A. (2020). Mild hyperlipidemia in mice aggravates platelet responsiveness in thrombus formation and exploration of platelet proteome and lipidome. Sci. Rep..

[6] Wang C., Lerner R.G., Frishman W.H. (2013). Statins and venous thromboembolic disease prophylaxis. Cardiol. Rev..

[7] Moosmann B., Behl C. (2004). Selenoprotein synthesis and side-effects of statins. Lancet.

[8] Zhao P., Tanaka T., Hirabayashi K., Zhang Y.J., Yang C.R., Kouno I. (2008). Caffeoylarbutin and related compounds from the buds of *Vaccinium dunalianum*. Phytochemistry.

[9] Luo X.L., Li N., Xu M., Zhu H.T., He P., Ding Y., Zhao P., Zhang Y.J. (2015). HPLC simultaneous determination of arbutin, chlorogenic acid and 6′-*O*-caffeoylarbutin in different parts of *Vaccinium dunalianum* Wight. Nat. Prod. Res..

[10] Xu M., Lao Q.C., Zhao P., Zhu X.Y., Zhu H.T., Luo X.L., Yang C.R., He J.H., Li C.Q., Zhang Y.J. (2014). 6′-*O*-Caffeoylarbutin inhibits melanogenesis in zebrafish. Nat. Prod. Res..

[11] Wang Y.P., Wang Y.D., Liu Y.P., Cao J.X., Yang M.L., Wang Y.F., Khan A., Zhao T.R., Cheng G.G. (2022). 6′-*O*-Caffeoylarbutin from Que Zui tea ameliorates acetaminophen-induced liver injury via enhancing antioxidant ability and regulating the PI3K signaling pathway. Food Funct..

[12] Adem Ş., Eyupoglu V., Sarfraz I., Rasul A., Zahoor A.F., Ali M., Abdalla M., Ibrahim I.M., Elfiky A.A. (2021). Caffeic acid derivatives (CAFDs) as inhibitors of SARS-CoV-2: CAFDs-based functional foods as a potential alternative approach to combat COVID-19. Phytomedicine.

[13] Hu X.Q., Qi C., Feng F., Wang Y., Di T.T., Meng Y.J., Wang Y.Z., Zhao N., Zhang X.W., Li P. (2022). Combining network pharmacology, RNA-seq, and metabolomics strategies to reveal the mechanism of *Cimicifugae Rhizoma*-*Smilax glabra Roxb* herb pair for the treatment of psoriasis. Phytomedicine.

[14] Oh K.K., Gupta H., Min B.H., Ganesan R., Sharma S.P., Won S.M., Jeong J.J., Lee S.B., Cha M.G., Kwon G.H. (2022). Elucidation of prebiotics, probiotics, postbiotics, and target from gut microbiota to alleviate obesity via network pharmacology study. Cells.

[15] Li S., Zhang B., Zhang N.B. (2011). Network target for screening synergistic drug combinations with application to traditional Chinese medicine. BMC Syst. Biol..

[16] Zon L.I., Peterson R.T. (2005). In vivo drug discovery in the zebrafish. Nat. Rev. Drug discov..

[17] Montalbano G., Mhalhel K., Briglia M., Levanti M., Abbate F., Guerrera M.C., D’Alessandro E., Laurà R., Germanà A. (2021). Zebrafish and flavonoids: Adjuvants against obesity. Molecules.

[18] Thomsen R., Christensen M.H. (2006). MolDock: A new technique for high-accuracy molecular docking. J. Med. Chem..

[19] Nickolas T.L., Radhakrishnan J., Appel G.B. (2003). Hyperlipidemia and thrombotic complications in patients with membranous nephropathy. Semin. Nephrol..

[20] McCrindle B.W. (2006). Hyperlipidemia in children. Thromb. Res..

[21] Grundy S.M. (1988). HMG-CoA reductase inhibitors for treatment of hypercholesterolemia. N. Engl. J. Med..

[22] Jing Y.S., Ma Y.F., Pan F.B., Li M.S., Zheng Y.G., Wu L.F., Zhang D.S. (2022). An insight into antihyperlipidemic effects of polysaccharides from natural resources. Molecules.

[23] Dini I., Mancusi A. (2023). Weight loss supplements. Molecules.

[24] Zhang J.K., Zhou X.L., Wang X.Q., Zhang J.X., Yang M.L., Liu Y.P., Cao J.X., Cheng G.G. (2022). Que Zui tea ameliorates hepatic lipid accumulation and oxidative stress in high fat diet induced nonalcoholic fatty liver disease. Food Res. Int..

[25] Yang J.H., Bai T.C., Shi L.L., Hou B., Tang R., Zhang R.P., Chen X.L. (2023). Antihyperlipidemic effect of *Vaccinium dunalianum* buds based on biological activity screening and LC-MS. J. Ethnopharmacol..

[26] Ganjali S., Blesso C.N., Banach M., Pirro M., Majeed M., Sahebkar A. (2017). Effects of curcumin on HDL functionality. Pharmacol. Res..

[27] Fang J., Little P.J., Xu S. (2018). Atheroprotective effects and molecular targets of tanshinones derived from herbal medicine danshen. Med. Res. Rev..

[28] Cao X.Y., Liao W., Xia H., Wang S.K., Sun G.J. (2022). The effect of resveratrol on blood lipid profile: A dose-response meta-analysis of randomized controlled trials. Nutrients.

[29] Berstein L.M. (2005). Clinical usage of hypolipidemic and antidiabetic drugs in the prevention and treatment of cancer. Cancer Lett..

[30] Koene R.J., Prizment A.E., Blaes A., Konety S.H. (2016). Shared risk factors in cardiovascular disease and cancer. Circulation.

[31] Nilsson S., Gustafsson J.A. (2002). Biological role of estrogen and estrogen receptors. Crit. Rev. Biochem. Mol. Biol..

[32] Murphy E. (2011). Estrogen signaling and cardiovascular disease. Circ. Res..

[33] Villablanca A., Lubahn D., Shelby L., Lloyd K., Barthold S. (2004). Susceptibility to early atherosclerosis in male mice is mediated by estrogen receptor alpha. Arterioscler. Thromb. Vasc. Biol..

[34] Wang Q., Du L.J., Hong J.N., Chen Z.L., Liu H.J., Li S.S., Xiao X., Yan S.K. (2021). Molecular mechanism underlying the hypolipidemic effect of Shanmei Capsule based on network pharmacology and molecular docking. Technol. Health Care.

[35] Moggs J.G., Orphanides G. (2001). Estrogen receptors: Orchestrators of pleiotropic cellular responses. EMBO Rep..

[36] Yang K., Zhang H.J., Luo Y., Zhang J.Y., Wang M., Liao P., Cao L., Guo P., Sun G.B., Sun X. (2017). Gypenoside XVII prevents atherosclerosis by attenuating endothelial apoptosis and oxidative stress: Insight into the ERα-mediated PI3K/Akt pathway. Int. J. Mol. Sci..

[37] Herrington D.M. (2003). Role of estrogen receptor-alpha in pharmacogenetics of estrogen action. Curr. Opin. Lipidol..

[38] Casazza K., Page G.P., Fernandez J.R. (2010). The association between the rs2234693 and rs9340799 estrogen receptor alpha gene polymorphisms and risk factors for cardiovascular disease: A review. Biol. Res. Nurs..

[39] Litwinoff E.M.S., del Pozo C.H., Ramasamy R., Schmidt A.M. (2015). Emerging targets for therapeutic development in diabetes and its complications: The RAGE signaling pathway. Clin. Pharmacol. Ther..

[40] Wu X.Z., Pan J.X., Yu J.J., Kang J., Hou S.Y., Cheng M.J., Xu L., Gong L.H., Li Y. (2023). DiDang decoction improves mitochondrial function and lipid metabolism via the HIF-1 signaling pathway to treat atherosclerosis and hyperlipidemia. J. Ethnopharmacol..

[41] Asadipooya K., Lankarani K.B., Raj R., Kalantarhormozi M. (2019). RAGE is a potential cause of onset and progression of nonalcoholic fatty liver disease. Int. J. Endocrinol..

[42] Ye J.H., Li L., Hu Z.X. (2021). Exploring the molecular mechanism of action of Yinchen Wuling powder for the treatment of hyperlipidemia, using network pharmacology, molecular docking, and molecular dynamics simulation. Bio. Med. Res. Int..

[43] Cai H., Griendling K.K., Harrison D.G. (2003). The vascular NAD(P)H oxidases as therapeutic targets in cardiovascular diseases. Trends Pharmacol. Sci..

[44] Yamagishi S., Nakamura K., Matsui T., Inagaki Y., Takenaka K., Jinnouchi Y., Yoshida Y., Matsuura T., Narama I., Motomiya Y. (2006). Pigment epithelium-derived factor inhibits advanced glycation end product-induced retinal vascular hyperpermeability by blocking reactive oxygen species-mediated vascular endothelial growth factor expression. J. Biol. Chem..

[45] Kamioka M., Ishibashi T., Sugimoto K., Uekita H., Nagai R., Sakamoto N., Ando K., Ohkawara H., Teramoto T., Maruyama Y. (2010). Blockade of renin-angiotensin system attenuates advanced glycation end products-mediated signaling pathways. J. Atheroscler. Thromb..

[46] Gut P., Reischauer S., Stainier D.Y.R., Arnaout R. (2017). Little fish, big data: Zebrafish as a model for cardiovascular and metabolic disease. Physiol. Rev..

[47] Ma W.J., Shi Y.L., Yang G.Z., Shi J., Ji J.P., Zhang Y., Wang J., Peng Q., Lin Z., Lv H. (2022). Hypolipidaemic and antioxidant effects of various Chinese dark tea extracts obtained from the same raw material and their main chemical components. Food Chem..

[48] Xiao Y., Huang Y.N., Long F.W., Yang D.M., Huang Y., Han Y.Y., Wu Y.P., Zhong K., Bu Q., Gao H. (2023). Insight into structural characteristics of theabrownin from Pingwu Fuzhuan brick tea and its hypolipidemic activity based on the in vivo zebrafish and in vitro lipid digestion and absorption models. Food Chem..

[49] Wang C.K., Cheng J., Liang X.G., Tan C., Jiang Q., Hu Y.Z., Lu Y.M., Fukunaga K., Han F., Li X.A. (2017). H_2_O_2_-responsive theranostic probe for endothelial injury imaging and protection. Theranostics.

[50] Zhu H., Lan C.H., Zhao D., Wang N., Du D., Luo H.B., Peng Z.F., Wang Y.M., Qiao Z.W., Huang Y. (2022). Wuliangye Baijiu but not ethanol reduces cardiovascular disease risks in a zebrafish thrombosis model. NPJ Sci. Food.

[51] Kim S., Chen J.T., Cheng T., Gindulyte A., He J., He S.Q., Li Q., Shoemaker B.A., Thiessen P.A., Yu B. (2023). PubChem 2023 update. Nucleic Acids Res..

[52] Daina A., Michielin O., Zoete V. (2019). SwissTargetPrediction: Updated data and new features for efficient prediction of protein targets of small molecules. Nucleic Acids Res..

[53] Keiser M.J., Roth B.L., Armbruster B.N., Ernsberger P., Irwin J.J., Shoichet B.K. (2007). Relating protein pharmacology by ligand chemistry. Nat. Biotechnol..

[54] Yao Z.J., Dong J., Che Y.J., Zhu M.F., Wen M., Wang N.N., Wang S., Lu A.P., Cao D.S. (2016). TargetNet: A web service for predicting potential drug-target interaction profiling via multi-target SAR models. J. Comput. Aided Mol. Des..

[55] Wang Y.Q., Wang Q.H., Huang H.Z., Huang W., Chen Y.X., McGarvey P.B., Wu C.H., Arighi C.N., UniProt Consortium (2021). A crowdsourcing open platform for literature curation in UniProt. PLoS Biol..

[56] Barshir R., Fishilevich S., Iny-Stein T., Zelig O., Mazor Y., Guan-Golan Y., Safran M., Lancet D. (2021). GeneCaRNA: A comprehensive gene-centric database of human non-coding RNAs in the GeneCards suite. J. Mol. Biol..

[57] Piñero J., Ramírez-Anguita J.M., Saüch-Pitarch J., Ronzano F., Centeno E., Sanz F., Furlong L.I. (2020). The DisGeNET knowledge platform for disease genomics: 2019 update. Nucleic Acids Res..

[58] Hamosh A., Amberger J.S., Bocchini C., Scott A.F., Rasmussen S.A. (2021). Online Mendelian inheritance in man (OMIM^®^): Victor McKusick’s magnum opus. Am. J. Med. Genet. A.

[59] Szklarczyk D., Gable A.L., Nastou K.C., Lyon D., Kirsch R., Pyysalo S., Doncheva N.T., Legeay M., Fang T., Bork P. (2021). The STRING database in 2021: Customizable protein–protein networks, and functional characterization of user-uploaded gene/measurement sets. Nucleic Acids Res..

[60] Zhang Q.W., Chen X.M., Hu Y.Y., Zhou T., Du M.H., Xu R., Chen Y.C., Tang P., Chen Z., Li J. (2023). BIRC5 inhibition is associated with pyroptotic cell death via caspase3-GSDME pathway in lung adenocarcinoma cells. Int. J. Mol. Sci..

[61] Sherman B.T., Hao M., Qiu J., Jiao X.L., Baseler M.W., Lane H.C., Imamichi T., Chang W. (2022). DAVID: A web server for functional enrichment analysis and functional annotation of gene lists (2021 update). Nucleic Acids Res..

[62] Berman H.M., Westbrook J., Feng Z., Gilliland G., Bhat T.N., Weissig H., Imamichi T., Chang W. (2000). The protein data bank. Nucleic Acids Res..

[63] O’Boyle N.M., Banck M., James C.A., Morley C., Vandermeersch T., Hutchison G.R. (2011). Open Babel: An open chemical toolbox. J. Cheminform..

[64] Morris G.M., Huey R., Lindstrom W., Sanner M.F., Belew R.K., Goodsell D.S., Olson A.J. (2009). AutoDock4 and AutoDockTools4: Automated docking with selective receptor flexibility. J. Comput. Chem..

[65] Lill M.A., Danielson M.L. (2011). Computer-aided drug design platform using PyMOL. J. Comput. Aided Mol. Des..

[66] Xiong G.L., Wu Z.X., Yi J.C., Fu L., Yang Z.J., Hsieh C., Yin M.Z., Zeng X.X., Wu C.K., Lu A.P. (2021). ADMETlab 2.0: An integrated online platform for accurate and comprehensive predictions of ADMET properties. Nucleic Acids Res..

